# Chemical Investigation and Dose-Response Phytotoxic Effect of Essential Oils from Two Gymnosperm Species (*Juniperus communis* var. *saxatilis* Pall. and *Larix decidua* Mill.)

**DOI:** 10.3390/plants11111510

**Published:** 2022-06-04

**Authors:** Sara Vitalini, Marcello Iriti, Valentina Vaglia, Stefania Garzoli

**Affiliations:** 1 Department of Food, Environmental and Nutritional Sciences, Milan State University, Via Mangiagalli 25, 20133 Milan, Italy; sara.vitalini@unimi.it; 2 National Interuniversity Consortium of Materials Science and Technology, Via G. Giusti 9, 50121 Firenze, Italy; marcello.iriti@unimi.it; 3 Department of Biomedical, Surgical and Dental Sciences, Milan State University, Via G. Celoria 2, 20133 Milan, Italy; 4 Department of Environmental Science and Policy, Milan State University, Via G. Celoria 2, 20133 Milan, Italy; valentina.vaglia@unimi.it; 5 Department of Drug Chemistry and Technology, Sapienza University, P.le Aldo Moro 5, 00185 Rome, Italy

**Keywords:** common juniper, common larch, Cupressaceae, Pinaceae, SPME-GC-MS, volatile compounds, herbicidal activity, weed control

## Abstract

The chemical composition of the liquid and vapor phases of leaf essential oils (EOs) obtained from two species of Gymnosperms (*Juniperus communis var. saxatilis* Willd. and *Larix decidua* Mill.) was investigated using the SPME-GC-MS technique. The results highlighted a composition characterized by 51 identified volatile compounds (34 in *J. communis* and 39 in *L. decidua*). In both bloils, monoterpenes prevailed over the sesquiterpenes, albeit with qualitative and quantitative differences. Sabinene (37.5% and 34.5%, respectively) represented the two most abundant components in the liquid and vapor phases of *J. communis*, and *α*-pinene (51.0% and 63.3%) was the main constituent in *L. decidua.* The phytotoxic activity of the two EOs was assessed in pre-emergence conditions using three concentrations in contact (2, 5, 10 µL/mL) and non-contact (2, 20, 50 µL) tests against *Lolium multiflorum* Lam. (Poaceae) and *Sinapis alba* L. (Brassicaceae). Treatments were effective in a dose-dependent manner by significantly reducing the germination (up to 100% and 45–60%, respectively, with filter paper and soil as a substrate) and the seedling development (1.3 to 8 times) of both target species. Moreover, an exploratory survey on the residual presence of volatile compounds in the soil at the end of the tests was carried out.

## 1. Introduction

Since man became a farmer 10,000 years ago, he has always had to fight against weeds, which have been a constant component of the agro-ecosystem. They have adapted to crop systems and co-evolved with them, significantly interfering with the human activities. From an ecological point of view, weeds are plants capable of colonizing potentially productive environments, managing to persist in conditions of repeated disturbance [1]. In the field, they cause significant damage for farmers. The most relevant effects include a decrease in crop production and a deterioration in its quality, in addition to an obstacle to mechanical operations. Another equally important aspect concerns the enrichment of the stock of seeds in the soil following the dissemination caused by their uncontrolled development [2]. Weeds can be controlled by various means (physical, ecological, mechanical, and chemical). Synthetic herbicides have been widely used since their discovery in the first decades of the previous century [3]. However, the growing problems related to weed management, such as resistance to herbicides, their low biodegradability, and high percolation and persistence in the soil, are increasing concerns relating to human health and environmental issues [4].

As a result of these problems, in recent years, various natural products have been studied for their allelopathic activity, including essential oils (EOs) and some of their constituents. EOs are multicomponent mixtures of plant volatiles able to exert a phytotoxic effect by providing an eco-chemical approach [5]. Their contact action causes rapid drying of the green plant parts by destroying the leaf cuticle and cell membranes [6]. In detail, allelochemicals can affect physiological functions, such as seed germination, respiration, photosynthesis, ion uptake, enzyme activity, transpiration, and hormone levels. They can also alter gene expression, the signal transduction chain, and permeability of the cell wall and membrane, and enhance the production of reactive oxygen species, or modify both the division and differentiation of cells [7]. The inhibition of germination and plant growth by EOs has been mainly attributed to terpenes, in particular, monoterpenes [8]. In general, essential oils offer an interesting class of compounds for management of parasites and weeds due to their low persistence in soil, relatively low toxicity towards mammals, and less stringent regulatory approval mechanisms [9].

In this work, we focused on the phytotoxic potential of EOs from two species of gymnosperms, namely *Juniperus communis* var. *saxatilis* Pall. (Cupressaceae) and *Larix decidua* Mill. (Pinaceae). The allelopathic activity of gymnosperms has long been known [10]. Several families—Araucariaceae, Cupressaceae, Pinaceae, Podocarpaceae, and Taxaceae—shows a strong negative allelopathic effect on the germination and growth of other plants [11]. In most of the cases, the allelochemicals identified as responsible for these interactions are the phenolic compounds leached from the litter consisting mainly of tree needles [10,11]. Until now, few reports have documented the phytotoxicity including autotoxicity of EOs obtained from leaves of species belonging to *Juniperus* [12,13,14] and *Larix* [15,16,17] genera. Our aim was to evaluate and compare the inhibitory effects of the two EOs used in different ways—via air and direct contact—on the germination and seedling growth of both monocot and dicot weed species, after determining the chemical composition of both liquid and vapor phases by means of the solid-phase micro-extraction gas chromatography-mass spectrometry (SPME-GC-MS) technique. The changes in the volatile profile occurring in the soil samples after the treatments with the EOs were also investigated.

## 2. Results

### 2.1. Essential Oil Chemical Composition

To describe the chemical profile of the liquid and vapor phases, the EOs were analyzed using the SPME-GC-MS technique. In total, 51 compounds were identified, of which 34 were in *J. communis* and 39 in *L. decidua* (Table 1). In both oils, the monoterpenes prevailed over the sesquiterpenes. Among the former, sabinene (37.5%, 34.5%) was the most abundant component in the liquid and vapor phases of *J. communis,* respectively, and α-pinene (51.0%, 63.3%) was the main constituent of *L. decidua*. Furthermore, the vapor phase of *J. communis* was enriched with limonene (14.0%), *p*-cymene (7.5%), and *β*-myrcene (12.6%), and that of *L. decidua* was enriched with *β*-ocimene (10.2%), *β*-pinene (7.9%), *β*-myrcene (6.2%) and limonene (4.5%) as principal compounds. 

Qualitative differences between the two EOs were noted. In particular, *β*-ocimene (10.2%), 1,8-cineole (3.2%), and trans-pinovcarveol (1.1%) were detected only in *L. decidua*; cis-thujopsene (2.1%), *δ*-cadinene (2.4%), *τ*-muurolol (0.6%), and *α*-cadinol (0.4%) were characteristic only in *J. communis*; and a number of other minor compounds (ranging from 0.1% to 0.3%) were detected in one of the EOs.

### 2.2. Soil Chemical Composition

The chemical composition of the EO residual vapor phase emitted from the soils at the end of both non-contact and contact tests was investigated using the SPME-GC/MS technique. The compounds detected in the samples with 20 or 50 µL of *J. communis* EO submitted to the non-contact test are listed in Table 2. In total, 55.9% of the starting compounds remained. cis-Thujopsene was the most abundant compound in all soils with percentage values equal to 30.2% and 40.5% in the samples where *L. multiflorum* seeds were sown at 20 and 50 µL, respectively, and 26.7% and 32.2% in the corresponding samples with *S. alba* seeds. This compound was followed by *α*-pinene (19.0% and 20.4%) in the presence of *L. multiflorum* and by *β*-elemene (12.9% and 13.7%) in the presence of *S. alba*. No residual volatile component was found in the soils treated with 2 µL of EOs.

The compounds released from the soils with 20 or 50 µL of *L. decidua* EO without direct contact with seeds are listed in Table 3. Among the five detected components, *α*-pinene was the most abundant in all the samples, with values ranging from 37.8% (soil with *S. alba* at 20 µL EO) to 87.7% (soil with *L. multiflorum* at 20 µL EO). Moreover, in this case, no residual volatile component was found to be emitted from the soils treated with 2 µL of EOs. 

Only three compounds were detected for the soil samples containing *L. multiflorum* and *S. alba* seeds in direct contact with 5 and 10 µL of *J. communis* and *L. decidua* EOs (Table 4 and Table 5). *α*-Thujene was the main volatile for the soils treated with *J. communis* EO, regardless of the type of seed species. In particular, it was the only one for the sample with *S. alba* seeds subjected to the action of 5 µL of *L. decidua* EO (Table 4). In contrast, *α*-pinene (≥81.8%) characterized the chemical composition of the volatile emission of the soils in direct contact with *L. decidua* EO (Table 5).

### 2.3. Effectiveness of EOs in Non-Contact Germination Test (Filter Paper Substrate)

The results for *J. communis* and *L. decidua* EOs showed a significant impact (*p*-values = 0.000) on all the considered indices, in both target species (Table 6). In particular, the treatments performed with the highest dose (50 µL) of *J. communis* EO totally inhibited the *L. multiflorum* germination (G = 0%) and reduced that of *S. alba* by 67.3%, also affecting the other considered parameters (CVG = −75%; MGT = +11.6%; SVI = −92.3%; root length = −90.3%; shoot length = −71.2%). The same dose of *Larix decidua* EO was even more effective, preventing the germination of both *L. multiflorum and S. alba*. Moreover, the obtained data evidenced that *L. multiflorum* was the most susceptible species. The values of its indices, except for MGT in some cases, were also significantly decreased by the lowest doses (2 and 20 µL) of both EOs.

### 2.4. Effectiveness of EOs in Non-Contact Germination Test (Soil Substrate)

The data reported in Table 7 also confirmed the efficacy of the *J. communis* and *L. decidua* EOs in the tests carried out using the soil as a substrate. All the indices, except G for *L. multiflorum* under the effect of *J. communis* EO, underwent significant variations (*p*-values < 0.05), if only due to the action of the highest tested dose. In detail, CVG of *L. multiflorum* and shoot length decreased by 49.5% and 33%, respectively, while MGT increased by 12.8% with 50 µL of *J. communis* EO. These values reached −60.8%, −59.4%, and +6.1% in the presence of the 50 µL of *L. decidua* EO. Regarding *S. alba*, the same treatments, respectively, reduced G by 26.6% and 43.2%, CVG by 32.4 and 60.2%, and shoot length by 25.5% and 42.6%, and increased MGT to +6.8% and +10.9%. In some cases, lower doses of both EOs were able to significantly affect the germination (e.g., −15% for *S. alba* at 2 µL of *J. communis* and −35.5% for *L. multiflorum* at 20 µL of *L. decidua*) and development (e.g., −9.9 for *L. multiflorum* at 2 µL of *J. communis* and −23.6% for *S. alba* at 20 µL of *L. decidua*) of the two target species.

The “interaction species × treatment” (EO doses) was not significant (*p*-value > 0.05) for G% after the *J. communis* treatment and for CVG and MGT indices after *L. decidua* use.

### 2.5. Effectiveness of EO in Contact Germination Test (Filter Paper Substrate)

The *J. communis* and *L. decidua* EOs tested in direct contact with seeds using filter paper as a substrate showed phytotoxic activity against both *L. multiflorum* and *S. alba,* influencing most of their germination and growth parameters (Table 8). In this case, the “interaction species × treatment” (EO doses) was not significant only for CVG (*p*-value > 0.05) after the *J. communis* treatments. 

Both EOs completely inhibited the germination of *L. multiflorum* (G = 0%) at the 50 µL dose. *L. decidua* also had the same effect at 20 µL, preventing the calculation of the related indices. At 2 µL, it inhibited G of *L. multiflorum* by 43.7%, CVG by 63%, SVI by 66.4%, and brought MGT to +5.8%. Its impact on *S. alba* was comparable (higher for some indices, lower for others) to that of *J. communis* when used at 50 µL (G, −74.5% vs. −53.2%; CVG, −83.2% vs. −69.6%; MGT, +11.1% vs. +16.3%, SVI, 91.3% vs. −75.4%, root length, −45.6% vs. −56.6%, shoot length, −21.8% vs. −39%), and was generally less effective at the two lower doses. 

### 2.6. Effectiveness of EO Vapor Phase in Contact Germination Test (Soil Substrate)

The data shown in Table 9 corroborated the above results regarding the effectiveness of the *J. communis* and *L. decidua* EOs against the two target species, despite the presence of the soil and the resulting interference. In general, they were able to similarly reduce the germination of *L. multiflorum* (by up to −53.9% and 50.2%, respectively). *J. communis* EO more influenced its CVG (−21.3% to −76.9%) and MGT (+8.3% to +16.7%) values than *L. decidua* EO. However, the latter limited the shoot elongation of *L. multiflorum* to 2.4 times compared to 1.3 times for *J. communis* EO. A similar trend was observed with respect to *S. alba* (G, −10.7% to −62.5% vs. −12.6% to −38.9%; CVG, −30.6% to −77.5% vs. −32.8% to −58.2%; MGT, +6.7% to 11.1% vs. +11.1% to +13.3%; shoot length, −0.7% to −35.3% vs. −17% to −40%).

Lastly, the “interaction species × treatment” (EO doses) was not significant for G and CVG after the *J. communis* EO treatment (*p*-value > 0.05), whereas it was significant only for the shoot length parameter in the presence of *L. decidua* EO (*p*-value = 0.00).

## 3. Discussion

The chemical composition of the liquid and vapor phases of two EOs obtained from leaves of *J. communis* var. *saxatilis* and *L. decidua* was determined by SPME-GC/MS analyses. Gymnosperms and, in particular, conifers produce EOs characterized by compounds belonging to the family of terpenes such as monoterpenes, sesquiterpenes, and their derivatives [18]. In agreement with previous works [19,20,21,22], our results showed monoterpenes prevail over sesquiterpenes. In particular, the two major components of *J. communis* and *L. decidua* EOs were *α*-pinene or sabinene, which were also found in other *Juniperus* [23,24] and *Larix* species [17,25,26]. Nevertheless, qualitative and/or quantitative differences in the chemical composition can be found, especially for minor compounds [27,28,29,30,31]. This is due to the different genotype or species [32,33], environmental conditions and soil composition [34,35], geographical area of origin [36] and harvesting period, in addition to different extraction methods and plant parts [37,38]. 

It is known that monoterpenes possess phytotoxic effects capable of leading to anatomical and physiological changes in plant seedlings, probably due to the inhibition of DNA synthesis or the rupture of mitochondrial membranes [39,40]. In particular, it was reported that *α*-pinene strongly inhibited mitochondrial ATP production [41] and root growth, also causing oxidative damage [42]. Furthermore, several monoterpenes, including α-pinene, have been shown to have inhibiting abilities on germination and radicle elongation of *Raphanus sativus* L. and *Lepidium sativum* L. [43]. Regarding sabinene, some studies documented the phytotoxicity of different EOs having this compound among the main constituents [44,45,46]. For example, a sabinene chemotype identified for EO from *Ravensara aromatica* Sonn. showed strong toxicity against *Oryza sativa* L. and *Lepidium sativum* L. [44]. Nonetheless, the higher percentage of *α*-pinene in our *L. decidua* EO may justify its greater effectiveness compared to the *J. communis* EO, in which sabinene was the most abundant. However, it is highly probable that the herbicidal activity of both EOs found in this work cannot be exclusively attributed to *α*-pinene and/or sabinene, but to the combined effect (synergistic or additive) of several molecules, including the minor ones. Indeed, as has been recently confirmed, mixtures of compounds are much more active and trigger different and more drastic responses [47].

EOs from conifer leaves have been reported to have high therapeutic potential [48] and, therefore, they are widely used in the treatment of infections and inflammatory phenomena [49]. Several studies demonstrated their biological properties [20,50,51,52,53,54,55,56,57], including allelopathic effects [10,11]. Nevertheless, EOs obtained from *Juniperus* and *Larix* species have been rather neglected from this point of view. Recently, Semerdjieva and co-authors [58] investigated the allelopathic activity of *J. sabina* L. and *J. excelsa* Bieb. EOs, reporting different inhibitory actions depending on the target species, the type of used EO, and the relative concentrations. Previously, Mehdizadeh et al. [14] documented the phytotoxic potential of EO obtained from the leaves of *J. polycarpos* var. *turcomanica* (B.Fedtsch.) R.P. Adams against three species of weeds, namely, *Portulaca oleracea* L., *Amaranthus retroflexus* L., and *Datura stramonium* L., attributing it to its major group of constituents, namely, monoterpenes hydrocarbons. Herbicidal effects were also reported for *J. oxycedrus* L. subsp. *macrocarpa* and *J. phoniceae* EOs, which were able to strongly reduce the germination and seedling growth of all tested weeds, in a dose-dependent manner [13,59]. In the case of *J. phoniceaea*, its EO also increased the proline level and caused severe electrolyte leakage from the roots of all target weeds, indicating membrane disruption and loss of integrity [59]. Finally, the *J. communis* EO exhibited no phytotoxic effect against *Ailanthus altissima* (Mill.) Swingle, resulting in 0% seedling mortality [23]. Few data are also available on the phytotoxicity of EOs from the genus *Larix*. The most recent work [17] studied the herbicidal effects of *L. kaempferi* (Lamb.) Carrière, demonstrating its capacity to inhibit the growth of *Brassica napus* L. by 50% in a seed bioassay and its inability to stop the development of new shoots after a foliar application of 10% EO in a greenhouse experiment. Previously, the negative effect of volatile substances of *L. gmelinii* (Rupr.) Kuzen. EO on the growth of *Fraxinus mandshurica* Rupr. was mainly attributed to *α*-pinene [15], while the EO from leaves and branches of *L. principis-rupprechtii* affected its own regeneration with significant inhibitory effects on the germination rate, radicle and hypocotyl length, and fresh mass [16]. 

In general, our data, in addition to highlighting a greater efficacy of the *L. decidua* EO, showed the different susceptibility of the two target species. *L. multiflorum* (monocotyledon) was more sensitive to treatments than *S. alba* (dicotyledon). Furthermore, the effects of both EOs were reduced by the interaction with the soil, with significant results still being obtained. In this type of substrate, we wanted to check for the possible presence of residual volatile terpenes. As expected, after 7 days, most of them were not detected, with differences between the two tests. Their absence, which may be due to the ability of soil particles to adsorb the volatile terpenes and subsequently release them to penetrate the seeds and exert their possible toxicity, deserves to be further investigated [60].

## 4. Materials and Methods

### 4.1. Plant Material

Bio EOs from leaves of *J. nana* and *L. decidua* were directly supplied from Bergila GmbH Srl (Falzes/Issengo-Bolzano, Italy) and stored at 4 °C until use.

Target seeds of *L. multiflorum* (grass) and *S. alba* L. (broadleaf) were provided by the organic farm “Terre di Lomellina” (Pavia, Italy) and purchased from the company “Padana Sementi” (Padua, Italy), respectively. Before use, they were sterilized with 1% sodium hypochlorite solution for 10 min, then repeatedly rinsed with distilled water.

### 4.2. Solid-Phase Microextraction (SPME)

To describe the chemical profile of the headspace from two EOs and of soil samples, a SPME device from Supelco (Bellefonte, PA, USA) was used for the sampling. The soil (~1 g) and the EOs (~2 mL) were individually placed into a 15 mL glass vial with PTFE-coated silicone septum. The chosen fiber was coated with 50/30μm DVB/CAR/PDMS (divinylbenzene/carboxen/polydimethylsiloxane). Before sampling, the fiber was conditioned at 270 °C for 20 min. First, the samples were equilibrated for 30 min at 50 °C prior to analysis. Subsequently, the fiber was exposed to the equilibrated headspace for 10 and 30 min to capture the volatile components from EOs and soil samples, respectively. Later, the fiber was inserted in a GC injector maintained at 250 °C for the desorption of collected components.

### 4.3. Gas Chromatography/Mass Spectrometry (GC/MS)

All analyses were performed using a Clarus 500 model Perkin Elmer (Waltham, MA, USA) gas chromatograph coupled with a mass spectrometer and equipped with an FID (flame detector ionization). In the GC oven was housed a Varian Factor Four VF-1 capillary column and helium was used as carrier gas at a flow rate of 1 mL/min. The adopted chromatographic conditions followed a previous study [61]. The mass spectra were obtained in the electron impact mode (EI), at 70 eV in scan mode in the range 35–400 *m*/*z*. The identification of volatile compounds was performed by matching their mass spectra with those stored in the Wiley 2.2 and Nist 02 mass spectra libraries database and by comparison of their linear retention indices (LRIs), relative to C_8_–C_25_ *n*-alkanes analyzed under the same conditions, with those available in the literature. Relative amounts of compounds, expressed as a percentage, were calculated in relation to the total area of the chromatogram by normalizing the peak area without the use of an internal standard and any factor correction. All analyses were carried out in triplicate.

### 4.4. Phytotoxic Studies

#### 4.4.1. Non-Contact Germination Test with EOs 

Seeds (15) of the target species *L. multiflorum* and *S. alba* were sown in 9 cm diameter Petri dishes lined with filter paper (Whatman No. 1) wetted with 4 mL of sterilized water. The EOs of *J. communis* or *L. decidua* were pipetted (2, 20, or 50 µL) into a small handmade aluminum container placed in the center of each Petri dish to avoid direct contact with seeds. To evaluate the phytotoxic activity of the EOs using a different substrate, the seeds (15) were also sown in 9 cm diameter Petri dishes filled with 25 g of non-fertilized soil (Vigorplant^®^ SER CA 98 V7, Fombio (Lo), Italy) wetted with 15 mL of sterilized water. Sterile 6 mm diameter disks (1 or 3) impregnated with different amounts (2, 20, or 50 µL) of *J. nana* or *L. decidua* EO were placed at the same depth as the seeds and covered with soil. In their respective controls, the EOs were absent and replaced by distilled water (2, 20 or 50 µL). Tests were carried out under a biological hood with vertical laminar flow. Subsequently, the suitably sealed (double layer of Parafilm) and initialed Petri dishes were incubated for 16 h light at 23 °C and 8 h darkness at 18 °C in a climatic chamber for 7 days. The experimental design included 3 quantities of each EO (treated samples) or distilled water (control samples) × 2 target species × 3 replicates × 2 runs.

#### 4.4.2. Contact Germination Test with EOs

Seeds (15) of the target species *L. multiflorum* and *S. alba* were sown in 9 cm diameter Petri dishes lined with filter paper (Whatman No. 1) wetted with 4 mL of an oily solution prepared with different concentrations (2, 5, and 10 µL/mL) of *J. communis* or *L. decidua* EO and using 0.1% Tween^®^ 20 (Sigma-Aldrich, Milan, Italy) as surfactant. To evaluate the same phytotoxic activity of the EOs using a different substrate, the seeds (15) were also sown in 9 cm diameter Petri dishes filled with 25 g of non-fertilized soil (Vigorplant^®^ SER CA 98 V7 Fombio (Lo), Italy) wetted with 15 mL of the same oily solutions prepared with different concentrations (2, 5, and 10 µL/mL) of *J. communis* or *L. decidua* EO and using 0.1% Tween^®^ 20 (Sigma-Aldrich, Milan, Italy) as surfactant. In their respective controls, the EOs were absent and replaced by 0.1% Tween^®^ 20 solution (4 or 15 mL). The test was carried out under a biological hood with vertical laminar flow. Subsequently, the suitably sealed (double layer of Parafilm) and initialed Petri dishes were incubated for 16 h light at 23 °C and 8 h darkness at 18 °C in a climatic chamber for 7 days. The experimental design included 3 quantities of each EO (treated samples) or distilled water (control samples) × 2 target species × 3 replicates × 2 runs.

### 4.5. Data Analysis

Phytotoxic effects of the *J. communis* and *L. decidua* EOs on germination and seedling development of the target species were described using the following indices:

1. Germination percentage (G) = Germinated seed number)/(Seed total number) × 100;
2. Coefficient of Velocity of Germination (CVG) = N1 + N2 + … + Ni/100 × N1T1 + … + NiTi, where N is the number of seeds germinated every day; T is the number of days from seeding corresponding to N [62];
3. Mean Germination Time (MGT) = (∑D × Germinated seed number)/(∑Germinated seed number), where D is the number of days from the beginning of germination, plus the number of seeds germinated on day D [63];
4. Seedling Vigor Index (SVI) = (Mean Root length + Mean Shoot length) × Germination %. [64].

The number of germinated seeds was detected every day for a week, and the measurements on the radicle and shoot of the seedlings were carried out at the end of the test, seven days after sowing.

### 4.6. Statistical Analysis

The data were evaluated with the support of IBM SPSS software, through the analysis of variance carried out separately for each EO (i.e., from the two species *J. communis* and *L. decidua*) and substrate (i.e., filter paper and soil). The germination and growth indices (i.e., G%, CVG, MGT, SVI, root length, shoot length) measured for the two target species (i.e., *L. multiflorum* and *S. alba*) under different treatments were taken into account as dependent variables.

The one-way ANOVA and the Turkey’s-b post hoc test were performed in order to establish the significant effect (at α ≤ 0.05) of the treatments with EOs (i.e., the different levels of concentration or quantity in EOs, respectively) on the target species and describe the homogenous subsets.

Moreover, the two-way ANOVA was performed, considering as factors the treatments with EOs and the species, in order to highlight the significant interaction (α ≤ 0.05) between “species × treatments” and then highlighting the species-specific effects of the treatments and the different behavior or susceptibility of *L. multiflorum* (grass) and *S. alba* (broadleaf).

## 5. Conclusions

Essential oils extracted from certain species of plants can represent a valid alternative to the use of synthetic chemicals as natural herbicidal agents capable of guaranteeing a phytotoxic effect but, at the same time, respectful of the environment and human health.

In our study, *J. communis* and *L. decidua* EOs were investigated in order to evaluate and compare their allelopathic effects, and on the basis of their chemical compositions. The findings showed that both EOs were active in a dose-dependent manner, but with greater efficacy shown in *L. decidua* EO against *Lolium multiflorum* and *Sinapis alba* L.

In conclusion, due to the obtained data, we can confirm that the EOs from gymnosperms, and their main components, may represent an important source for the development of new low-impact natural products against weeds.

## Figures and Tables

**Table 1:** Chemical composition (percentages mean values ± standard deviation) of EO liquid and vapor phases.

N°	Component ^1^	LRI ^2^	LRI ^3^	*Juniperus communis* EO ^4^	*Juniperus communis* EO ^5^	*Larix decidua* EO ^6^	*Larix decidua* EO ^7^
1	*α*-thujene	821	823	4.2 ± 0.02	4.9 ± 0.06	0.3 ± 0.02	0.5 ± 0.03
2	*α*-pinene	942	943	19.0 ± 0.04	10.3 ± 0.03	51.0 ± 0.05	63.3 ± 0.03
3	camphene	945	946	0.2 ± 0.02	-	1.4 ± 0.02	1.2 ± 0.01
4	dehydrosabinene	960	956	-	-	-	0.4 ± 0.02
5	sabinene	976	972	37.5 ± 0.03	34.5 ± 0.02	0.8 ± 0.02	1.0 ± 0.03
6	*β*-pinene	985	978	3.0 ± 0.04	12.6 ± 0.03	2.3 ± 0.02	7.9 ± 0.02
7	*β*-myrcene	990	987	1.1 ± 0.03	-	9.7 ± 0.03	6.2 ± 0.02
8	*α*-phellandrene	1007	1005	0.7 ± 0.03	1.4 ± 0.03	0.3 ± 0.03	0.2 ± 0.07
9	3-carene	1010	1008	-	0.9 ± 0.03	-	-
10	*α*-terpinene	1012	1010	2.4 ± 0.06	5.8 ± 0.03	-	-
11	*p*-cymene	1020	1016	1.2 ± 0.02	7.5 ± 0.05	1.1 ± 0.03	1.1 ± 0.01
12	limonene	1026	1023	5.8 ± 0.03	14.0 ± 0.07	3.9 ± 0.06	4.5 ± 0.02
13	*β*-ocimene	1029	1024	-	-	10.2 ± 0.04	10.2 ± 0.02
14	1,8-cineole	1030	1025	-	-	3.2 ± 0.06	1.9 ± 0.05
15	*γ*-terpinene	1053	1054	5.0 ± 0.04	1.0 ± 0.02	0.5 ± 0.03	0.3 ± 0.02
16	terpinolene	1082	1080	2.6 ± 0.03	4.3 ± 0.06	1.0 ± 0.04	0.5 ± 0.03
17	p-cymenene	1093	1091	-	-	0.3 ± 0.02	0.1 ± 0.01
18	*α*-campholenal	1127	1125	-	-	0.3 ± 0.03	-
19	trans-pinocarveol	1137	1134	-	-	1.1 ± 0.02	0.3 ± 0.03
20	pinocarvone	1149	1145	-	-	0.2 ± 0.02	-
21	borneol	1160	1155	-	-	0.2 ± 0.03	-
22	terpinen-4-ol	1165	1160	4.2 ± 0.02	2.7 ± 0.02	0.8 ± 0.02	0.1 ± 0.02
23	*α*-terpineol	1182	1183	0.2 ± 0.01	-	0.4 ± 0.02	-
24	carveol	1202	1201	-	-	-	0.1 ± 0.02
25	cuminal	1215	1211	-	-	0.1 ± 0.01	-
26	phellandral	1255	1249	-	-	0.1 ± 0.04	-
27	bornyl acetate	1294	1290	0.3 ± 0.02	-	2.6 ± 0.07	0.1 ± 0.01
28	4-terpinenyl acetate	1307	1304	0.1 ± 0.01	-	0.8 ± 0.02	-
29	*α*-terpinyl acetate	1336	1333	0.4 ± 0.03	-	-	-
30	*α*-cubebene	1352	1348	0.2 ± 0.03	-	-	-
31	copaene	1390	1385	0.3 ± 0.02	-	0.1 ± 0.02	-
32	*β*-elemene	1408	1406	1.8 ± 0.05	-	0.1 ± 0.02	-
33	longifolene	1410	1408	-	-	0.2 ± 0.02	tr
34	*β*-caryophyllene	1427	1424	1.3 ± 0.03	-	2.2 ± 0.07	tr
35	cis-thujopsene	1438	1435	2.1 ± 0.03	-	-	-
36	humulene	1471	1465	0.8 ± 0.04	-	0.7 ± 0.03	-
37	*γ*-muurolene	1490	1486	0.5 ± 0.02	-	0.3 ± 0.02	-
38	germacrene D	15,001	1500	0.1 ± 0.02	-	0.5 ± 0.05	-
39	*α*-muurolene	1507	*	1.0 ± 0.02	-	0.5 ± 0.02	-
40	guaia-1(10), 11-diene	1509	1505	0.3 ± 0.02	-	-	-
41	*δ*-cadinene	1533	1530	2.4 ± 0.03	-	-	-
42	*α*-cadinene	1539	*	-	-	0.1 ± 0.02	-
43	*α*-calacorene	1541	1539	-	-	0.1 ± 0.02	-
44	spathulenol	1576	1571	0.1 ± 0.02	-	-	-
45	caryophyllene oxide	1616	1613	0.1 ± 0.02	-	0.2 ± 0.02	-
46	epicubenol	1620	1618	0.1 ± 0.02	-	-	-
47	humulene epoxide II	1622	*	-	-	0.1 ± 0.02	-
48	*δ*-cadinol	1627	*	-	-	0.2 ± 0.01	-
49	*τ*-cadinol	1630	1625	-	-	0.1 ± 0.02	-
50	*τ*-muurolol	1641	1639	0.6 ± 0.02	-	-	-
51	*α*-cadinol	1678	1676	0.4 ± 0.03	-	-	-
	SUM			100.0	99.9	97.9	99.9
	Terpenoids			87.6	99.9	89.6	99.7
	Sesquiterpenoids			11.8	-	5.1	-
	Others			0.6	-	3.2	0.2

^1^ The components are reported according to their elution order on apolar column; ^2^ Linear Retention indices measured on apolar column; ^3^ Linear Retention indices from literature; * LRI not available; ^4^ Percentage values of *J. communis* liquid phase components; ^5^ Percentage values of *J. communis* vapor phase components; ^6^ Percentage values of *L. decidua* liquid phase components; ^7^ Percentage values of *L. decidua* vapor phase components; - Not detected; tr: traces (mean value < 0.1%).

**Table 2:** Chemical composition (percentage mean values ± standard deviation) of soil samples with *L. multiflorum* and *S. alba* seeds in non-direct contact with *J. communis* EO.

N°	Component ^1^	LRI ^2^	LRI ^3^	Soil ^4^	Soil ^5^	Soil ^6^	Soil ^7^
1	*α*-thujene	821	823	4.2 ± 0.02	0.1 ± 0.02	4.0 ± 0.03	3.7 ± 0.03
2	*α*-pinene	942	943	19.0 ± 0.05	0.4 ± 0.02	20.4 ± 0.02	1.2 ± 0.02
3	Sabinene	976	972	2.5 ± 0.02	-	3.2 ± 0.02	3.2 ± 0.02
4	*α*-terpinene	1012	1010	5.1 ± 0.02	0.3 ± 0.02	4.5 ± 0.02	6.0 ± 0.06
5	*p*-cymene	1020	1016	9.0 ± 0.02	0.6 ± 0.03	4.8 ± 0.04	6.5 ± 0.03
6	Limonene	1026	1023	4.9 ± 0.02	0.3 ± 0.02	2.9 ± 0.02	4.6 ± 0.02
7	*γ*-terpinene	1053	1054	6.3 ± 0.02	0.3 ± 0.04	6.5 ± 0.02	7.8 ± 0.02
8	Terpinolene	1082	1080	2.2 ± 0.02	1.0 ± 0.06	-	-
9	*α*-cubebene	1352	1348	-	2.3 ± 0.02	0.5 ± 0.03	2.1 ± 0.01
10	Copaene	1390	1385	0.3 ± 0.02	2.8 ± 0.02	0.9 ± 0.02	3.2 ± 0.02
11	*β*-elemene	1408	1406	9.7 ± 0.02	12.9 ± 0.02	7.1 ± 0.02	13.7 ± 0.02
12	*β*-caryophyllene	1427	1424	1.6 ± 0.02	4.2 ± 0.03	2.1 ± 0.02	4.4 ± 0.01
13	cis-thujopsene	1438	1435	30.2 ± 0.02	26.7 ± 0.04	40.5 ± 0.03	32.2 ± 0.02
14	Humulene	1471	1465	1.5 ± 0.02	2.1 ± 0.02	1.0 ± 0.05	3.0 ± 0.02
15	*γ*-muurolene	1490	1486	-	4.9 ± 0.04	-	-
17	*α*-muurolene	1507	*	-	1.9 ± 0.04	-	-
16	*δ*-cadinene	1533	1530	0.6 ± 0.04	10.6 ± 0.02	0.3 ± 0.02	2.6 ± 0.01
18	*τ*-muurolol	1641	1639	-	0.9 ± 0.02	-	-
19	*α*-cadinol	1678	1676	2.9 ± 0.02	27.7 ± 0.04	1.2 ± 0.02	5.6 ± 0.02
	SUM			100.0	100.0	99.9	99.8
	Terpenoids			53.2	3.0	46.3	33.0
	Sesquiterpenoids			46.5	94.2	52.7	63.6
	Others			0.3	2.8	0.9	3.2

^1^ The components are reported according to their elution order on apolar column ^2^ Linear Retention indices measured on apolar column; ^3^ Linear Retention indices from literature; * LRI not available; ^4^ Percentage mean values of the volatiles from soil with 20 µL of *J. communis* EO and *L. multiflorum* seeds; ^5^ Percentage values of the volatiles from soil with 20 µL of *J. communis* EO and *S. alba* seeds; ^6^ Percentage mean values of the volatiles from soil with 50 µL of *J. communis* EO and *L. multiflorum* seeds; ^7^ Percentage mean values of the volatiles from soil with 50 µL of *J. communis* EO and *S. alba* seeds; - Not detected.

**Table 3:** Chemical composition (percentages mean values ± standard deviation) of soil samples with *L. multiflorum* and *S. alba* seeds in non-contact with *L. decidua* EO.

N°	Component ^1^	LRI ^2^	LRI ^3^	Soil ^4^	Soil ^5^	Soil ^6^	Soil ^7^
1	*α*-pinene	942	943	87.7 ± 0.05	37.8 ± 0.03	72.5 ± 0.03	66.7 ± 0.05
2	*β*-pinene	985	978	4.6 ± 0.02	3.3 ± 0.02	2.8 ± 0.03	7.1 ± 0.02
3	*β*-myrcene	990	987	7.7 ± 0.02	10.2 ± 0.03	12.6 ± 0.03	19.1 ± 0.02
4	*β*-ocimene	1029	1024	-	13.8 ± 0.03	7.7 ± 0.02	4.6 ± 0.02
5	*β*-caryophyllene	1427	1424	-	34.9 ± 0.04	4.4 ± 0.02	2.5 ± 0.03
	SUM			100.0	100.0	100.0	100.0

^1^ The components are reported according to their elution order on apolar column ^2^ Linear Retention indices measured on apolar column; ^3^ Linear Retention indices from literature; ^4^ Percentage mean values of the volatiles from soil with 20 µL of *L. decidua* EO and *L. multiflorum* seeds; ^5^ Percentage mean values of the volatiles from soil with 20 µL of *L. decidua* EO and *S. alba* seeds; ^6^ Percentage mean values of the volatiles from soil with 50 µL of *L. decidua* EO and *L. multiflorum* seeds; ^7^ Percentage mean values of the volatiles from the soil with 50 µL of *L. decidua* EO and *S. alba* seeds; - Not detected.

**Table 4:** Chemical composition (percentages mean values ± standard deviation) of soil samples with *L. multiflorum* and *S. alba* seeds in contact with *J. communis* EO.

N°	Component ^1^	LRI ^2^	LRI ^3^	Soil ^4^	Soil ^5^	Soil ^6^	Soil ^7^
1	*α*-thujene	821	823	-	100.0 ± 0.02	42.2 ± 0.03	87.9 ± 0.03
2	*β*-elemene	1408	1406	-	-	17.4 ± 0.02	12.1 ± 0.02
3	cis-thujopsene	1438	1435	-	-	40.4 ± 0.03	-
	SUM				100.0	100.0	100.0

^1^ The components are reported according to their elution order on apolar column ^2^ Linear Retention indices measured on apolar column; ^3^ Linear Retention indices from literature; ^4^ Percentage mean values of the volatiles from soil with 5 µL of *J. communis* EO and *L. multiflorum* seeds; ^5^ Percentage mean values of the volatiles from soil with 5 µL of *J. communis* EO and *S. alba* seeds; ^6^ Percentage mean values of the volatiles from soil with 10 µL of *J. communis* EO and *L. multiflorum* seeds; ^7^ Percentage mean values of the volatile from soil with 10 µL of *J. communis* EO and *S. alba* seeds; - Not detected.

**Table 5:** Chemical composition (percentages mean values ± standard deviation) of soil samples with *L. multiflorum* and *S. alba* seeds in direct contact with *L. decidua* EO.

N°	Component ^1^	LRI ^2^	LRI ^3^	Soil ^4^	Soil ^5^	Soil ^6^	Soil ^7^
1	*α*-pinene	942	943	96.4 ± 0.05	93.7 ± 0.03	88.9 ± 0.03	81.8 ± 0.05
2	*β*-pinene	985	978	3.6 ± 0.02	4.7 ± 0.02	11.1 ± 0.03	18.2 ± 0.02
3	*β*-myrcene	990	987	-	1.6 ± 0.03	-	-
	SUM			100.0	100.0	100.0	100.0

^1^ The components are reported according to their elution order on apolar column ^2^ Linear Retention indices measured on apolar column; ^3^ Linear Retention indices from literature; ^4^ Percentage mean values of the volatiles from soil with 5 µL of *L. decidua* EO and *L. multiflorum* seeds; ^5^ Percentage mean values of the volatiles from soil with 5 µL of *L. decidua* EO and *S. alba* seeds; ^6^ Percentage mean values of the volatiles from soil with 10 µL of *L. decidua* EO and *L. multiflorum* seeds; ^7^ Percentage mean values of the volatiles from soil with 10 µL of *L. decidua* EO and *S. alba* seeds; - Not detected.

**Table 6:** Germination and growth values of two target species (*Lolium multiflorum* and *Sinapis alba*) under the phytotoxic effects of different doses of *Juniperus communis* var. *saxatilis* and *Larix decidua* EOs using filter paper as a substrate.

Target Species	EO (µL)	G (%)	CVG	MGT	SVI	Root (mm)	Shoot (mm)
***Juniperus communis*** **var. *saxatilis***							
*Lolium* *multiflorum*	0	93.3 ± 5.3 a	89.8 ± 9.0 a	5.1 ± 0.1 a	11,064 ± 306 a	72.3 ± 6.5 a	46.6 ± 2.0 a
	2	80.3 ± 9.4 b	67.7 ± 6.6 b	5.1 ± 0.1 a	5129 ± 720 b	41.5 ± 4.5 b	22.3 ± 3.2 b
	20	35.0 ± 8.5 c	21.5 ± 7.0 c	5.4 ± 0.2 b	772 ± 314 c	15.7 ± 3.6 c	5.8 ± 0.7 c
	50	0.0 ± 0.0 d	n.d.	n.d.	n.d.	n.d.	n.d.
	*F*	154.856	157.288	1721.960	580.626	214.413	476.953
	*p*-value	0.000 *	0.000 *	0.000 *	0.000 *	0.000 *	0.000 *
*Sinapis* *alba*	0	83.3 ± 8.7 a	102.6 ± 13.9 a	4.3 ± 0.1 a	4522 ± 301 a	30.9 ± 2.5 a	23.6 ± 1.6 a
	2	78.5 ± 8.3 a	89.9 ± 13.1 a	4.3 ± 0.1 a	3711 ± 354 b	29.3 ± 3.2 a	18.1 ± 1.6 b
	20	70.0 ± 8.5 a	85.7 ± 15.8 a	4.4 ± 0.1 a	2195 ± 120 c	18.6 ± 3.3 b	13.0 ± 0.9 c
	50	35.0 ± 6.3 b	26.8 ± 3.0 b	4.8 ± 0.2 b	349 ± 99 d	3.0 ± 0.6 c	6.8 ± 0.9 d
	*F*	29.702	28.903	9.183	225.386	96.659	121.614
	*p*-value	0.000	0.000	0.002	0.000	0.000	0.000
**Interaction species × treatment**							
	*F*	20.398	10.168	749.786	209.782	68.815	150.270
	*p*-value	0.000 *	0.000 *	0.000 *	0.000 *	0.000 *	0.000 *
***Larix decidua***							
	0	90.0 ± 3.5 a	92.0 ± 3.2 a	5.0 ± 0.2 a	10,450 ± 116 a	68.6 ± 1.4 a	47.5 ± 3.4 a
*Lolium* *multiflorum*	2	63.0 ± 8.2 b	46.8 ± 6.9 b	5.2 ± 0.1 ab	2662 ± 590 b	20.0 ± 1.6 b	21.9 ± 3.1 b
	20	54.0 ± 15.4 b	28.3 ± 9.9 c	5.5 ± 0.3 b	907 ± 366 c	3.6 ± 1.3 c	13.0 ± 1.5 c
	50	0.0 ± 0.0 c	n.d.	n.d.	n.d.	n.d.	n.d.
	*F*	71.962	151.869	814.006	731.639	2514.839	275.249
	*p*-value	0.000 *	0.000 *	0.000 *	0.000 *	0.000 *	0.000 *
*Sinapis* *alba*	0	83.3 ± 11.6 a	106.0 ± 17.6 a	4.3 ± 0.1 a	3440 ± 891 a	20.0 ± 4.3 a	20.9 ± 3.5 a
	2	81.8 ± 6.7 a	101.0 ± 13.9 a	4.4 ± 0.0 b	2734 ± 537 a	18.2 ± 5.4 a	15.5 ± 2.2 b
	20	71.8 ± 11.5 a	72.3 ± 16.1 b	4.8 ± 0.0 c	2444 ± 525 a	17.5 ± 0.9 a	16.4 ± 2.3 b
	50	0.0 ± 0.0 b	n.d.	n.d.	n.d.	n.d.	n.d.
	*F*	81.159	50.067	6953.429	26.377	28.495	59.168
	*p*-value	0.000 *	0.000 *	0.000 *	0.000 *	0.000 *	0.000 *
**Interaction species × treatment**							
	*F*	4.164	11.159	15.836	127.252	215.139	63.298
	*p*-value	0.017 *	0.000 *	0.000 *	0.000 *	0.000 *	0.000 *

Values are mean ± standard deviation; asterisk and different letters indicate statistically significant differences at *p*-value ≤ 0.05 among treatments in each species. *F*-value and *p*-value of the ANOVA test. Abbreviations: G%, Germination percentage; CVG, Coefficient of Velocity of Germination; MGT, Mean Germination Time, SVI, Seedling Vigor Index.

**Table 7:** Germination and growth values of two target species (*Lolium multiflorum* and *Sinapis alba*) under the phytotoxic effects of different doses of *Juniperus communis* var. *saxatilis* and *Larix decidua* EOs using soil as a substrate.

Target Species	EO (µL)	G (%)	CVG	MGT	Shoot (mm)
***Juniperus communis*** **var. *saxatilis***					
*Lolium* *multiflorum*	0	86.8 ± 5.3	92.0 ± 5.8 a	4.7 ± 0.1 a	73.1 ± 2.2 a
	2	78.3 ± 6.7	82.0 ± 11.6 a	4.8 ± 0.1 a	66.6 ± 2.8 b
	20	76.5 ± 4.0	71.5 ± 5.3 a	5.0 ± 0.2 b	65.6 ± 3.6 b
	50	63.3 ± 20.1	46.5 ± 19.2 b	5.3 ± 0.0 c	49.0 ± 4.9 c
	*F*	3.073	10.652	19.886	34.017
	*p*-value	0.069	0.001 *	0.000 *	0.000 *
*Sinapis* *alba*	0	88.5 ± 3.0 a	101.5 ± 6.5 a	4.4 ± 0.1 a	29.8 ± 1.1 a
	2	75.3 ± 9.9 b	75.2 ± 11.2 b	4.4 ± 0.1 ab	29.4 ± 1.3 a
	20	65.0 ± 6.3 b	72.5 ± 1.9 b	4.6 ± 0.1 ab	24.5 ± 1.6 b
	50	65.0 ± 6.3 b	68.6 ± 5.5 b	4.7 ± 0.1 b	22.2 ± 1.4 b
	*F*	10.649	18.279	5.742	30.647
	*p*-value	0.001 *	0.000 *	0.011 *	0.000 *
**Interaction species × treatment**					
	*F*	0.921	3.165	4.787	15.100
	*p*-value	0.446	0.043 *	0.009 *	0.000 *
***Larix decidua***					
*Lolium* *multiflorum*	0	88.0 ± 10.0 a	93.3 ± 12.4 a	4.9 ± 0.1 a	72.1 ± 2.8 a
	2	86.5 ± 7.5 a	82.3 ± 16.1 a	5.0 ± 0.1 a	69.3 ± 2.8 a
	20	56.8 ± 8.7 b	55.2 ± 8.1 b	4.9 ± 0.0 a	44.4 ± 3.6 b
	50	48.3 ± 5.5 b	36.6 ± 6.0 b	5.2 ± 0.2 b	29.3 ± 3.2 c
	*F*	25.388	20.468	5.965	176.381
	*p*-value	0.000 *	0.000 *	0.010 *	0.000 *
*Sinapis* *alba*	0	76.5 ± 7.0 a	80.5 ± 11.3 a	4.6 ± 0.1 a	30.5 ± 1.1 a
	2	76.8 ± 8.7 a	77.8 ± 10.5 a	4.6 ± 0.0 a	31.3 ± 0.7 a
	20	68.5 ± 3.0 a	68.5 ± 6.2 a	4.7 ± 0.1 a	23.3 ± 2.0 b
	50	43.5 ± 7.0 b	32.0 ± 4.9 b	5.1 ± 0.1 b	17.5 ± 2.0 c
	*F*	21.634	27.273	30.000	69.892
	*p*-value	0.000 *	0.000 *	0.000 *	0.000 *
**Interaction species × treatment**					
	*F*	4.055	2.357	0.583	66.368
	*p*-value	0.018 *	0.097	0.632	0.000 *

Values are mean ± standard deviation; asterisk and different letters indicate statistically significant differences at *p*-value ≤ 0.05 among treatments in each species. *F*-value and *p*-value of the ANOVA test. Abbreviations: G%, Germination percentage; CVG, Coefficient of Velocity of Germination; MGT, Mean Germination Time.

**Table 8:** Germination and growth values of two target species (*Lolium multiflorum* and *Sinapis alba*) under the phytotoxic effects of different doses of *Juniperus communis* var. *saxatilis* and *Larix decidua* EOs using filter paper as a substrate.

Target Species	EO (µL/mL)	G (%)	CVG	MGT	SVI	Root (mm)	Shoot (mm)
***Juniperus communis*** **var. *saxatilis***							
*Lolium* *multiflorum*	0	90.0 ± 3.5 a	84.8 ± 4.6 a	5.0 ± 0.1 a	7037 ± 568 a	42.9 ± 3.3 a	35.2 ± 0.5 a
	2	53.5 ± 13.0 b	37.1 ± 14.7 b	5.4 ± 0.1 b	2632 ± 678 b	34.9 ± 2.9 b	14.4 ± 3.8 b
	5	31.8 ± 13.9 c	17.8 ± 8.9 c	5.4 ± 0.2 b	1020 ± 660 c	20.4 ± 6.7 c	9.3 ± 2.0 c
	10	0.0 ± 0.0 d	n.d.	n.d.	n.d.	n.d.	n.d.
	*F*	61.330	68.439	2630.647	126.727	88.602	187.572
	*p*-value	0.000 *	0.000 *	0.000 *	0.000 *	0.000 *	0.000 *
*Sinapis* *alba*	0	81.8 ± 6.7 a	103.8 ± 9.4 a	4.3 ± 0.1 a	2780 ± 361 a	16.8 ± 1.9 a	17.2 ± 1.7 a
	2	49.8 ± 8.3 b	41.9 ± 9.9 b	4.5 ± 0.2 ab	1686 ± 326 b	20.6 ± 1.9 b	13.2 ± 0.7 b
	5	39.8 ± 13.5 b	35.2 ± 3.7 b	4.9 ± 0.3 b	705 ± 165 c	7.7 ± 1.2 c	10.4 ± 2.1 b
	10	38.3 ± 3.5 b	31.6 ± 16.9 b	5.0 ± 0.2 b	683 ± 155 c	7.3 ± 2.3 c	10.5 ± 1.1 b
	*F*	21.253	37.024	7.728	55.029	51.470	17.225
	*p*-value	0.000 *	0.000 *	0.004 *	0.000 *	0.000 *	0.000 *
**Interaction species × treatment**							
	*F*	10.297	2.403	460.778	48.706	39.572	79.502
	*p*-value	0.000 *	0.092	0.000 *	0.000 *	0.000 *	0.000 *
***Larix decidua***							
	0	91.5 ± 3.0 a	84.0 ± 8.3 a	5.2 ± 0.1 a	8338 ± 714 a	49.4 ± 3.6 a	41.7 ± 3.9 a
*Lolium* *multiflorum*	2	51.5 ± 12.8 b	31.2 ± 8.9 b	5.5 ± 0.1 b	2227 ± 824 b	16.6 ± 5.7 b	36.2 ± 8.2 b
	5	0.0 ± 0.0 c	n.d.	n.d.	n.d.	n.d.	n.d.
	10	0.0 ± 0.0 c	n.d.	n.d.	n.d.	n.d.	n.d.
	*F*	183.326	169.513	15,155.667	208.653	191.104	214.739
	*p*-value	0.000 *	0.000 *	0.000 *	0.000 *	0.000 *	0.000 *
*Sinapis* *alba*	0	71.8 ± 6.2 a	76.3 ± 6.6 a	4.5 ± 0.2 a	2892 ± 288 a	16.9 ± 1.3 a	23.4 ± 1.8 a
	2	66.5 ± 12.2 a	62.4 ± 21.3 a	4.9 ± 0.2 b	2029 ± 461 b	14.0 ± 2.5 a	16.3 ± 1.3 ab
	5	64.8 ± 9.9 a	56.3 ± 11.1 a	5.0 ± 0.1 b	1610 ± 315 b	9.7 ± 2.4 b	15.3 ± 2.1 b
	10	18.3 ± 6.7 b	12.8 ± 5.5 b	5.0 ± 0.2 b	252 ± 108 c	9.2 ± 1.9 b	18.3 ± 6.7 ab
	*F*	29.871	18.409	7.446	47.666	12.202	3.851
	*p*-value	0.000 *	0.000 *	0.004 *	0.000 *	0.001 *	0.038 *
**Interaction species × treatment**							
	*F*	38.219	14.891	1426.056	96.108	99.856	60.698
	*p*-value	0.000*	0.000 *	0.000 *	0.000 *	0.000 *	0.000 *

Values are mean ± standard deviation; asterisk and different letters indicate statistically significant differences at *p*-value ≤ 0.05 among treatments in each species. *F*-value and *p*-value of the ANOVA test. Abbreviations: G%, Germination percentage; CVG, Coefficient of Velocity of Germination; MGT, Mean Germination Time, SVI, Seedling Vigor Index.

**Table 9:** Germination and growth values of two target species (*Lolium multiflorum* and *Sinapis alba*) under the phytotoxic effects of different doses of *Juniperus communis* var. *saxatilis* and *Larix decidua* EOs using soil as a substrate.

Target Species	EO (µL/mL)	G (%)	CVG	MGT	Shoot (mm)
***Juniperus communis*** **var. *saxatilis***					
*Lolium* *multiflorum*	0	98.3 ± 3.5 a	112.8 ± 11.5 a	4.8 ± 0.1 a	71.7 ± 3.2 a
	2	83.5 ± 4.0 b	88.8 ± 9.4 b	4.8 ± 0.1 a	69.6 ± 3.6 a
	5	56.5 ± 13.8 c	47.5 ± 18.3 c	5.2 ± 0.0 a	66.9 ± 1.3 a
	10	45.3 ± 3.5 c	26.0 ± 9.2 c	5.6 ± 0.4 b	43.0 ± 8.7 b
	*F*	41.002	38.913	14.510	28.223
	*p*-value	0.000 *	0.000 *	0.000 *	0.000 *
*Sinapis* *alba*	0	93.3 ± 5.5 a	115.2 ± 13.7 a	4.5 ± 0.1 a	28.3 ± 0.5 a
	2	83.5 ± 4.0 ab	80.0 ± 4.7 b	4.8 ± 0.1 b	28.1 ± 3.6 a
	5	70.0 ± 14.1 b	63.2 ± 13.6 b	5.0 ± 0.0 c	22.4 ± 1.9 b
	10	35.0 ± 12.3 c	25.7 ± 11.0 c	5.0 ± 0.1 c	18.3 ± 1.4 c
	*F*	26.273	43.250	25.826	19.860
	*p*-value	0.000 *	0.000 *	0.000 *	0.000 *
**Interaction species × treatment**					
	*F*	2.655	1.436	4.688	11.654
	*p*-value	0.071	0.257	0.010 *	0.000 *
***Larix decidua***					
*Lolium* *multiflorum*	0	90.0 ± 8.5 a	102.3 ± 19.0 a	4.7 ± 0.0 a	72.1 ± 3.4 a
	2	71.8 ± 9.9 b	68.0 ± 3.3 b	4.9 ± 0.0 ab	59.6 ± 2.8 b
	20	71.5 ± 3.0 b	57.1 ± 16.0 bc	5.2 ± 0.5 ab	57.0 ± 2.8 b
	50	44.8 ± 9.9 c	35.0 ± 14.6 c	5.4 ± 0.1 c	29.5 ± 11.2 c
	*F*	19.881	15.100	4.696	33.525
	*p*-value	0.000 *	0.000 *	0.022 *	0.000 *
*Sinapis* *alba*	0	81.8 ± 3.5 a	96.0 ± 9.5 a	4.5 ± 0.1 a	31.2 ± 2.1 a
	2	71.5 ± 8.3 ab	64.5 ± 11.1 ab	5.1 ± 0.1 b	25.9 ± 2.2 b
	20	58.3 ± 17.3 ab	48.5 ± 25.2 b	5.1 ± 0.3 b	20.4 ± 0.7 c
	50	50.0 ± 16.7 c	40.1 ± 19.1 b	5.0 ± 0.1 b	18.7 ± 2.1 c
	*F*	4.799	8.060	8.892	36.371
	*p*-value	0.020 *	0.003 *	0.002 *	0.000 *
**Interaction species × treatment**					
	*F*	1.155	1.140	1.387	17.195
	*p*-value	0.347	0.353	0.271	0.000 *

Values are mean ± standard deviation; asterisk and different letters indicate statistically significant differences at *p*-value ≤ 0.05 among treatments in each species. *F*-value and *p*-value of the ANOVA test. Abbreviations: G%, Germination percentage; CVG, Coefficient of Velocity of Germination; MGT, Mean Germination Time.

## Data Availability

All generated data are included in this article.
